# Systems Pharmacological Approach of* Pulsatillae Radix* on Treating Crohn's Disease

**DOI:** 10.1155/2017/4198035

**Published:** 2017-06-01

**Authors:** Su Yeon Suh, Won G. An

**Affiliations:** Department of Pharmacology, School of Korean Medicine, Pusan National University, Yangsan, Gyeongnam 50612, Republic of Korea

## Abstract

In East Asian traditional medicine,* Pulsatillae Radix* (PR) is widely used to treat amoebic dysentery and renowned for its anti-inflammatory effects. This study aimed to confirm evidence regarding the potential therapeutic effect of PR on Crohn's disease using a system network level based in silico approach. Study results showed that the compounds in PR are highly connected to Crohn's disease related pathways, biological processes, and organs, and these findings were confirmed by compound-target network, target-pathway network, and gene ontology analysis. Most compounds in PR have been reported to possess anti-inflammatory, anticancer, and antioxidant effects, and we found that these compounds interact with multiple targets in a synergetic way. Furthermore, the mRNA expressions of genes targeted by PR are elevated significantly in immunity-related organ tissues, small intestine, and colon. Our results suggest that the anti-inflammatory and repair and immune system enhancing effects of PR might have therapeutic impact on Crohn's disease.

## 1. Introduction

Inflammatory bowel disease (IBD) may be categorized clinically as Crohn's disease or ulcerative colitis [1]. Crohn's disease usually causes a variety of systemic symptoms, which include chronic inflammation of the bowel [2]. Although any part of the digestive tract from mouth to anus may be affected, usually the small intestine (ileum) and the large intestine (colon) are involved; ileocolic Crohn's accounts for 50% of cases, ileal Crohn's for 30%, and colic Crohn's for 20% of cases [3]. Symptoms vary though its common manifestations include persistent diarrhea, rectal bleeding, abdominal cramps, and pain, though fever, extreme fatigue, and weight loss are also common [1, 2, 4]. Constipation is also a frequent symptom and can lead to bowel obstruction and, thus, increase the risk of bowel cancer. Complications involving other than the gastrointestinal (GI) tract include anemia, arthritis, liver disease, eye inflammation, and skin rashes [2, 4].

According to a statistical report issued by the Health Insurance Review and Assessment Service in Korea in 2016, the number of Crohn's disease patients increased from 13,920 in 2011 to 18,332 in 2015, an average annual increase of 7.1%. Furthermore, over the same period, total medical cost has increased by 19.4% annually, and more than half of patients are in 20s or 30s. A systematic review about the economic and quality-of-life burden of Crohn's disease reported that, in the USA, Germany, France, UK, Italy, and Spain, in all countries combined, Crohn's medical costs totaled €30 billion annually and that patient quality of life was substantially diminished by the physical, emotional, and social effects of the disease [5].

Although Crohn's disease is a global health problem [5], its pathology remains poorly understood [1, 6]. Nevertheless, it has been established that its etiology is associated with complex interactions between environmental, immune, microbial, and genetic factors [4, 7], though a number of authors have suggested that the primary defect in Crohn's disease is one of relative immunodeficiency [6, 8, 9].

A systematic review of publications from 1947 to 2013 involving controlled studies of herbal therapies in IBD indicated that at least 50 percent of IBD patients used some form of complementary and alternative medicine (CAM), since most herbal therapies had been reported to have anti-inflammatory effects and plausible action mechanisms in IBD with minimal adverse effects [10]. Moreover, herbal medicines are being increasingly used and requested by IBD patients not only in Asia, but also in Western countries [11].

Of the anti-inflammatory herbal medicines,* Pulsatillae Radix* (Baekduong, PR) is worth considering as a potential treatment for Crohn's disease as it was widely used in traditional medicine to treat amoebic dysentery and has also been demonstrated scientifically to have anti-inflammatory effects experimentally [12, 13]. PR is traditional Korean herbal medicine prepared from the roots of* Pulsatilla koreana Nakai* and contains several phytochemicals, including anemonin, hederagenin, oleanolic acid, and deoxypodophyllotoxin [14, 15]. Some experimental study results have shown that PR has various biological activities. For instance, aqueous and ethanol extracts of PR have been reported to demonstrate anticancer effects in anaplastic thyroid cancer [16], methanol extract of PR was found to have anti-inflammatory effects in lipopolysaccharide (LPS) exposed rats [17], and PR has also been reported to inhibit adipocyte differentiation and to suppress adipogenesis [13].

According to the meridian tropism theory of traditional medicine, the effects of PR reach to the stomach meridian and the large intestine meridian, which is in accord with the organs commonly affected by Crohn's disease. In terms of the selection of traditional medication, meridian tropism theory plays an important role, as it is one of the core principles of traditional medicine [18]. Based on meridian tropism theory, each herbal medicine possesses different affinities for certain organs and meridians of the body [19].

**Figure 1:**
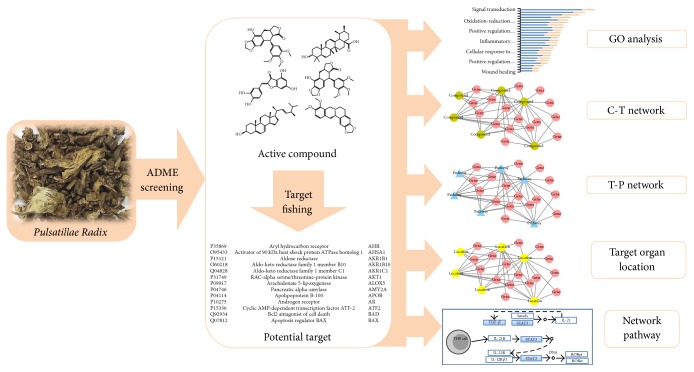
Workflow: network analysis, relevant organ location network analysis, gene ontology, and pathway analysis were performed on active compounds identified in* Pulsatillae Radix* (PR) by screening and target fishing.

In the present study, we sought to confirm the therapeutic effects of PR in Crohn's disease using system level analysis and a network based in silico approach. A schematic of the network pharmacological study is summarized in Figure 1.

## 2. Material and Methods

### 2.1. Identification of Active Compounds

According to the Traditional Chinese Medicine Systems Pharmacology (TCMSP, http://ibts.hkbu.edu.hk/LSP/tcmsp.php) database (a free phytochemical database of herbal medicine), PR contains 57 identified compounds. Parameters related to absorption, distribution, metabolism, and excretion (ADME), namely, human drug-likeness (DL) [20], oral bioavailability (OB) [21], and Caco-2 permeability (Caco-2) [22], were employed to filter out potential active compounds.

#### 2.1.1. Drug-Likeness Evaluation

DL helps filter out “drug-like” compounds in oriental herbs, as DL represents a qualitative concept for valuations based on how “drug-like” prospective compound is [23]. Accordingly, a high DL may lead to a greater possibility of therapeutic success, and compounds with a higher DL value are more likely to possess certain biological properties [24]. Calculations of DL in the TCMSP database are based on the Tanimoto coefficient formula [25] as follows:

(1) $$\begin{array}{c} F\left(\;A,B\;\right)=\frac{A\times B}{A^{2}+B^{2}-A\times B}, \end{array}$$

where *A* represents the molecular parameters of herbal compounds and *B* is the average molecular parameter of all compounds in the DrugBank database (http://www.drugbank.ca/) [26]. In the present study, we excluded compounds with a DL value of < 0.18.

#### 2.1.2. Oral Bioavailability (OB) Prediction

Oral bioavailability (OB) is defined as the absorption ratio of an active compound into the systemic circulation, which represents convergence of the ADME process [21]. OB values are dependent on drug dissolution in the gastrointestinal (GI) tract, intestinal and hepatic first-pass metabolisms, and intestinal membrane permeation, and, thus, OB is considered a major pharmacokinetic parameter for drug evaluations [24]. In this study, the OB threshold was set as ≥ 15%.

#### 2.1.3. Caco-2 Permeability Screening

Caco-2 permeability is used to predict the absorption of an orally administered drug [22]. Surface absorptivity of the small intestine is maximized by villi and microvilli; for this reason, orally administered drugs are mostly absorbed in the small intestine [27]. Moreover, the movement of orally administered drugs across the intestinal epithelial barrier determines the rate and extent of human absorption and ultimately affects drug bioavailability [28]. In the present study, compounds with OB, DL, and Caco-2 values of >15%, >0.18, and >−0.4, respectively, were regarded as active and subjected to analysis. In addition, we included some compounds with lower ADME profile than above thresholds, for the reason that those were reported to possess anti-inflammatory, antioxidant, anticancer, and antibacterial effects. This study was about only one single herb, and for this reason we did not use a high threshold of ADME profile to filter potential active compounds. Instead, we lowered the standard of OB in order to analyze the most potential targets of PR.

### 2.2. Target Fishing

Molecular targets of filtered potential active compounds were found in the TCMSP [29], Similarity Ensemble Approach (SEA, http://sea.bkslab.org), and the Binding Database (http://www.bindingdb.org). In addition, filtered compound-target interaction profile mapping was performed using the UniProt database (http://www.uniprot.org/) [30].

### 2.3. Gene Ontology (GO) Analysis

Biological process (BP) of gene ontology (GO) analysis was employed to determine the biological properties of target genes [31]. GO annotation provides statistical analyses on gene function information. In this research, GO BP terms with *P* values < 0.01 were employed and the data was collected using the DAVID 6.8 Gene Functional Classification Tool (http://david.abcc.ncifcrf.gov/).

### 2.4. Network Construction and Analysis

In order to understand the multiscale interactions between the active compounds of PR and targets, two types of networks were built: (1) a compound-target network (C-T network), in which nodes represented either compounds or target proteins, and edges indicated compound-target connections; and (2) a target-pathway network (T-P network), which was used to extract pathways from the KEGG database (http://www.genome.jp/kegg/) and to filter out terms highly associated with Crohn's disease and *P* values of < 0.01. Related targets were mapped onto relevant pathways to produce the T-P network. Both networks were generated in Cytoscape 3.4.0, an open-source biological network visualization and data integration software package [32].

### 2.5. Target Organ Location Network

Tissue-specific patterns of mRNA expression can indicate important associations with particular biological events or gene functions [33]. Thus, to explore the beneficial effects of PR on Crohn's disease, it was important to determine the tissue mRNA expression profiles of target proteins at the organ level [34]. The target organ location network was used with the Dataset: GeneAtlas U133A, gcrma (http://biogps.org). The BioGPS database provides expression data acquired by direct measurements of gene expressions by microarrays analysis [35]. First, the mRNA expression patterns of each target gene in 84 parts of organ tissues were obtained. Second, average values were calculated for each gene. Third, above average mRNA expressions in relevant organ tissues were extracted and arranged by frequency. Finally, a target organ location network was constructed using Cytoscape 3.4.0 and organ-specific, Crohn's disease related, gene overexpression data.

### 2.6. GEO2R Analysis

Using Gene Expression Omnibus (GEO, https://www.ncbi.nlm.nih.gov/geo), we compared mRNA expression pattern of normal groups and Crohn's disease groups. GEOquery and limma R packages of GEO2R tool were employed to identify highly expressed genes.

### 2.7. Network Pathway

In order to elucidate the action mechanisms of PR in Crohn's disease, filtered target proteins were input into the pathway map of inflammatory bowel disease acquired from the Kyoto Encyclopedia of Genes and Genomes (KEGG, http://www.kegg.jp/) database.

## 3. Results

### 3.1. Identification of Active Compounds

Of the 57 compounds (as shown in Supplementary Table S1 in Supplementary Material available online at https://doi.org/10.1155/2017/4198035) in PR acquired from the TCMSP, excluding compounds with no target information, 19 compounds with a known target met the criteria OB ≥ 15%, Caco-2 ≥ −0.4, and DL ≥ 0.18. Additionally 13 compounds reported to have anti-inflammatory, antioxidant, anticancer, and antibacterial effects were added, and finally 32 compounds were analyzed (as shown in Table 1).

A number of these 32 compounds have been shown experimentally to have various biological activities. For example, antioxidative effect of cernuoside (C12; DL = 0.79, OB = 2.69, Caco-2 = −1.51) was experimentally identified [36]. Pinoresinol (C23; DL = 0.52, OB = 4.25, Caco-2 = 0.52) was reported to have anti-inflammatory properties [37]. *β*-Sitosterol (C32; DL = 0.71, OB = 5.84, Caco-2 = 1.42) and campesterol (C23; DL = 0.72, OB = 5.57, Caco-2 = 1.6) were reported to have the protecting effect by natural and synthetic antioxidants during heating [38]. Antiallergic effects of scoparone (C25; DL = 0.09, OB = 74.75, Caco-2 = 0.85) was experimentally demonstrated in rat model, which attenuates IgE-mediated allergic response in mast cells [39]. Aureusidin (C7; DL = 0.24, OB = 53.42, Caco-2 = 0.07) was reported to have marked antioxidant activity and to be useful for the treatment of several diseases [40, 41], and anemosapogenin (C5; DL = 0.77, OB = 17.87, Caco-2 = 0.07) has antitumor effects [42, 43]. Betulonic acid (C9; DL = 0.78, OB = 16.83, Caco-2 = 0.65) possesses various medical effects, such as antiviral (HIV-1), anticancer, and immunomodulatory activities [44]. Cauloside (C11; DL = 0.4, OB = 6.84, Caco-2 = −0.82) from blue cohosh was reported to inhibit proinflammatory cytokine induction by LPS [45]. Dauricine (C13; DL = 0.9, OB = 23.65, Caco-2 = 0.37) from Asiatic moonseed was reported to have significant antibacterial and anti-inflammatory effects [46], and ergosterol (C14; DL = 0.72, OB = 14.29, Caco-2 = 1.47) [47] from the mushroom and isorhamnetin (C17; DL = 0.31, OB = 49.6, Caco-2 = 0.31) [48] were both found to have anti-inflammatory effects. Furthermore, oleanolic acid (C21; DL = 0.76, OB = 29.02, Caco-2 = 0.59) and ursolic acids (C30; DL = 0.75, OB = 16.77, Caco-2 = 0.67) have been reported to have antioxidative and anti‐inflammatory effect [49, 50]. As mentioned above, PR contains many compounds, which are ubiquitous in plants, herbs, and fruits, with anti-inflammatory, anticancer, and antioxidative effects.

### 3.2. Target Fishing

These 32 identified active compounds interact with 182 target proteins (Table 2); that is, on average, they interact with 5.7 target genes, which does much to explain the polypharmacological and synergistic effects of PR on multiple targets [51].

### 3.3. GO Analysis

**Figure 2:**
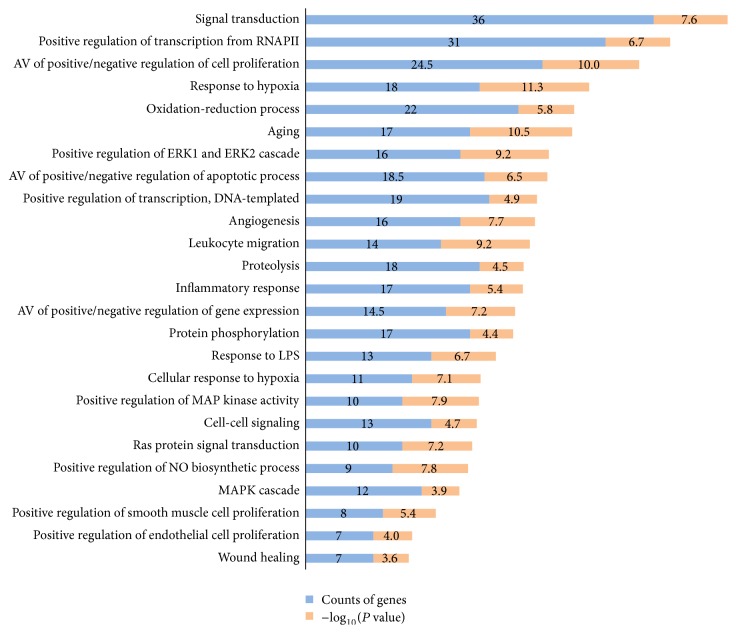
GO analysis: 25 biological process (BP) of gene ontology (GO) terms sorted by *P* value < 0.01. Counts of genes and *P* value related to each BP terms are shown. The *y*-axis represents BP terms for the target genes, and the *x*-axis shows counts of genes and −log⁡_10_(*P* value).

For the filtered 182 target genes, 469 biological process terms with *P* values of < 0.01 were sorted using the functional annotation chart of the DAVID 6.8 Gene Functional Classification Tool and *P* values were adjusted using Benjamini-Hochberg method. This process resulted in the identification of 25 biological process terms. GO analysis showed that the 182 genes were highly related to inflammation, proliferation, oxidation reduction, and the regulations of apoptosis and signal transduction (Figure 2).

In detail, phosphatidylinositol-4, 5-bisphosphate 3-kinase catalytic subunit, gamma isoform (PIK3CG), interleukin-6 (IL6), tumor necrosis factor (TNF), C-C motif chemokine 2 (CCL2), prostaglandin E2 receptor EP3 subtype (PTGER3), oxidized low-density lipoprotein receptor 1 (OLR1), prostaglandin G/H synthase 2 (PTGS2), and others are related to “inflammatory response.”

Androgen receptor (AR), interleukin-6 (IL6), heparin-binding growth factor 2 (FGF2), GTPase HRas (HRAS), hematopoietic cell protein-tyrosine phosphatase (PTPN6), and signal transducer and activator of transcription 3 (STAT3) are related to both the “positive regulation and negative regulation of cell proliferation.”

Xanthine dehydrogenase/oxidase (XDH), 5,6-dihydroxyindole-2-carboxylic acid oxidase (TYRP1), prostaglandin G/H synthase 2 (PTGS2), neutrophil cytosol factor 1 (NCF1), lanosterol 14-alpha demethylase (CYP51A1), amine oxidase [flavin-containing] A (MAOA), dual oxidase 2 (DUOX2), and others are associated with “oxidation-reduction process.”

Bcl-2-like protein 1 (BCL2L1), interleukin-6 (IL6), mitogen-activated protein kinase 8 (MAPK8), and cellular tumor antigen p53 (TP53) are associated with both the “positive regulation and negative regulation of apoptotic process.”

To summarize, it is likely that the therapeutic effect of PR in Crohn's disease is due to its anti-inflammatory and repair process and immune system enhancing effects.

### 3.4. Network Construction and Analysis

**Figure 3:**
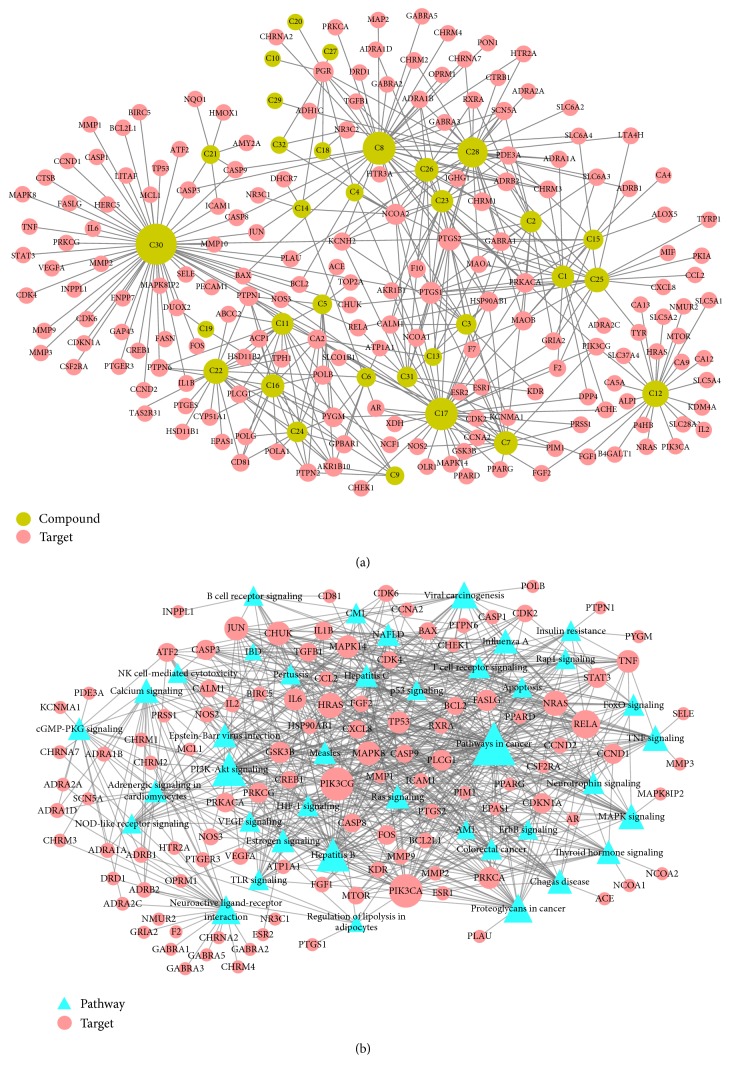
C-T network and T-P network: (a) is a compound-target (C-T) network, and nodes represent compounds and targets; and (b) shows a target-pathway (T-P) network, and circular nodes represent compounds and triangles pathways. Node size is related to the degree and edges demonstrate interactions between nodes.

To visualize more conveniently the multitargeted effects of PR, network analysis was used to investigate its actions within the context of the whole human genome [52, 53]. As shown in Figure 3, constructed (A) C-T and (B) T-P network demonstrated multicompound and multitargeted effects and relations between various pathways and targets. Circular nodes represent compounds and targets in the C-T network and triangles and circular nodes show pathways and compounds in the T-P network. In both networks, node size was regulated by degree centrality and edges showed interactions between nodes.

The C-T network showed 415 interactions between the 182 targets and 32 active compounds of PR. Ursolic acid (C30, degree = 55) had the highest number of interactions with targets, followed by beta-sitosterol (C8, degree = 37) and isorhamnetin (C17, degree = 36), and, thus, these results demonstrated that single molecules can target multiple receptors [54]. Likewise, prostaglandin G/H synthase 2 (PTGS2, degree = 15) displayed the most affinitive connections with compounds, followed by prostaglandin G/H synthase 1 (PTGS1, degree = 13) and nuclear receptor coactivator 2 (NCOA2, degree = 11). According to betweenness centrality results, protein-tyrosine phosphatase 1B (PTPN1, betweenness = 0.11) was second highest followed by PTGS2 (betweenness = 0.12). 28 (88%) Of the 32 active compounds were connected with more than two targets and 86 (47%) of the 182 targets interact with more than one compound. This network analysis results clearly demonstrated the multitargeting natures of herbal compounds; besides it showed that ursolic acid (C30) is the most essential compound in PR.

In addition, 40 pathways related to Crohn's disease were extracted to construct the T-P network. According to degree centrality, “pathways in cancer” (degree = 49) had the highest number of connections with the targets, followed by the “PI3K-Akt signaling pathway” (degree = 34) and “hepatitis B” (degree = 33). In the same manner, phosphatidylinositol 4,5-bisphosphate 3-kinase catalytic subunit alpha isoform (PIK3CA, degree = 33), phosphatidylinositol 4,5-bisphosphate 3-kinase catalytic subunit gamma isoform (PIK3CG, degree = 33), and nuclear factor NF-*κ*B p65 subunit (RELA, degree = 25) demonstrate higher affinitive connections with pathways. Betweenness centrality and degree centrality results were similar; there was only little difference. “Pathways in cancer” (betweenness = 0.19) had the highest betweenness among the targets, which concurred with degree centrality, and this was followed by “neuroactive ligand-receptor interaction” (betweenness = 0.15) and “PI3K-Akt signaling pathway” (betweenness = 0.10). Regarding highest betweenness targets, PIK3CA (betweenness = 0.10), PIK3CG (betweenness = 0.10), and prostaglandin E2 receptor EP3 subtype (PTGER3, betweenness = 0.04) showed most affinitive connections with pathways.

### 3.5. Target Organ Location Network

**Figure 4:**
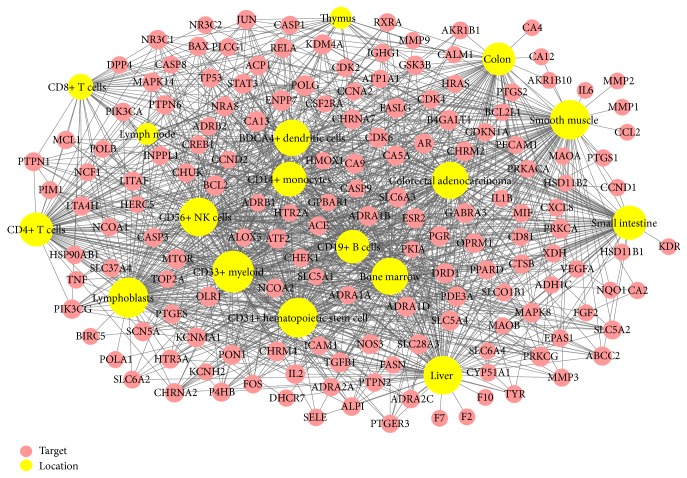
Target organ location network: the tissue-specific patterns of the mRNA expressions of targets in organs related to the immune system, colon, and small intestine. Nodes represent targets and organ locations. Node size is relative to the degree. Gene expression data was based on gene expression microarrays analysis results in BioGPS.

The tissue mRNA expression profiles of target genes at the organ level were investigated to identify effects of PR on Crohn's disease. No mRNA expression information of three of genes, muscarinic acetylcholine receptor M1 (CHRM1), G-protein coupled receptor TGR-1 (NMUR2), and taste receptor type 2 member 31 (TAS2R31), was found in the BioGPS. In total, the mRNA expression profiles of 179 of 182 genes were analyzed (Supplementary Table S2). 158 Of the genes displayed above average mRNA expressions in 17 relevant organ tissues, namely, in BDCA4+ dendritic cells, bone marrow, CD14+ monocytes, CD19+ B cells, CD33+ myeloid cells, CD34+ hematopoietic stem cells, CD4+ T cells, CD56+ NK cells, CD8+ T cells, colon, colorectal adenocarcinoma, liver, lymph nodes, lymphoblasts, small intestine, smooth muscle, and thymus. In addition, these 17 organ tissues, retina, prefrontal cortex, pineal, amygdala, cardiac myocyte, heart, whole blood, and other tissues, were also associated with relatively high mRNA expressions. Networks of the tissue mRNA expressions of 158 target genes and PR compounds are shown in Figure 4, nodes represent organs and genes, and node sizes indicate the number of interactions between nodes.

In detail, among 84 organ tissues, CD33+ myeloid showed the most overexpressed mRNA expression, 104 genes of 158 target genes were overexpressed in CD33+ myeloid, followed by 97 genes in lymphoblasts, 95 in each of smooth muscle and CD34+ hematopoietic stem cells, 91 genes in liver and CD56+ NK cell, 84 in bone marrow and colorectal adenocarcinoma, 82 in BDCA4+ dendritic cells, 75 in CD14+ monocytes, 73 in small intestine, 72 in CD4+ T cells, 70 in CD19+ B cells, 67 in colon, 38 in CD8+ T cells, 23 in thymus, and 22 in lymph nodes. It is evident that most genes were overexpressed in several organs at the same time.

Additionally, intestinal alkaline sphingomyelinase (ENPP7), DNA polymerase catalytic subunit (POLG), and carbonic anhydrase 13 (CA13) recorded beyond average mRNA expressions in all 17 organs. Furthermore, more than 146 (92%) of 158 target genes were overexpressed in two or more organ tissues, suggesting that these organs and compounds in PR are closely related. Furthermore, because the above 17 organs are highly related to immunity, our study results indicate that the therapeutic effects of PR on Crohn's disease are due to its targeting and activating the immune system.

The other 21 target genes, such as acetylcholinesterase (ACHE), pancreatic alpha-amylase (AMY2A), and muscarinic acetylcholine receptor M3 (CHRM3), did not show above average mRNA expression in the 17 organs.

### 3.6. GEO2R Analysis

Comparison data between normal tissue and Crohn's disease patients' mRNA expression pattern from Gene Expression Omnibus (GEO) was collected. We employed GEOquery and limma R packages of GEO2R tool to identify highly expressed genes in 6 datasets. Dataset accession numbers are as follows: GSE24287, GSE60083, GSE6731, GSE36807, GSE68570, and GSE72780. To sum up, 86 normal samples and 149 Crohn's disease samples were analyzed in each dataset. GEO2R presented the top 250 highly expressed genes in Crohn's disease group compared to the control group and we deleted overlaps, so, in the end, 1182 genes were sorted out.

We found out that there were 23 common genes (Table 3) between target genes of PR and highly expressed genes of Crohn's disease dataset from GEO.

### 3.7. Network Pathway

**Figure 5:**
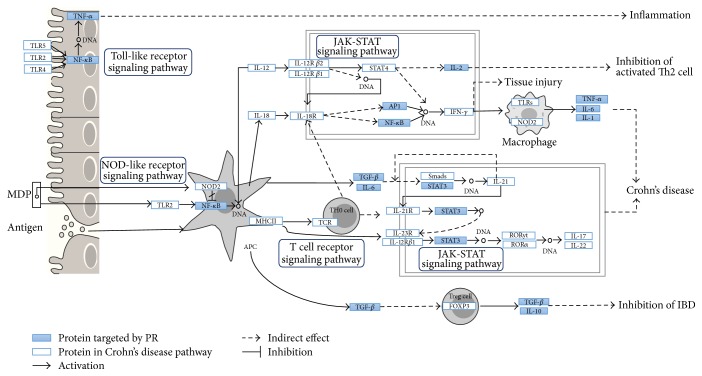
Network pathway: pathway enrichment analysis was performed. 182 filtered target genes were mapped into the Crohn's disease pathway from the Kyoto Encyclopedia of Genes and Genomes (KEGG) to confirm the possible effect pathway of* Pulsatillae Radix* (PR) on Crohn's disease.

In order to investigate further the effect of PR in Crohn's disease, we performed pathway enrichment analysis (Figure 5). Using the IBD pathway provided by the KEGG pathway database, we confirmed the pathway mapping effect of PR in Crohn's disease by inputting the filtered human target genes into the pathway. The KEGG pathway was constructed according to the current knowledge of the pathogenesis IBD.

The synthesis of inflammatory cytokines, such as IL-1, IL-6 and TNF-*α*, is mediated by NF-*κ*B, which is a key regulator of inflammation [55, 56]. We found that oleanolic acid derivative (C22) targets IL-1; ursolic acid (C30) targets all of IL-1, IL-6, and TNF; isorhamnetin (C17), scoparone (C25), and ursolic acid (C30) target nuclear factor NF-*κ*B p65 subunit (RELA); and scoparone (C25) and ursolic acid (C30) target NF-*κ*B inhibitor alpha (CHUK), which suggests that these compounds affect NF-*κ*B activity. Cernuoside (C12) targets IL-2. Beta-sitosterol (C8) targets transforming growth factor beta-1 (TGFB1) and transcription factor AP-1 (JUN), and ursolic acid (C30) also targets transcription factor AP-1 (JUN) and signal transducer and activator of transcription 3 (STAT3). Furthermore, NF-*κ*B and AP-1 in combination are highly related to the initial inflammatory response and to the development of acquired immunity [57]. Moreover, IL-6-mediated STAT3 activation on mucosal T cells may has been suggested to play a role in the development of IBD [58].

## 4. Discussion

In this study, a network pharmacology method with active compounds filtration, multiple drug target prediction, gene ontology, network analysis, relevant organ location network, and pathway enrichment analysis were employed to determine the targets of PR in relation to Crohn's disease. Our study shows that PR is highly connected to the pathways, biological processes, and organs of Crohn's disease. A pharmacological approach was used to identify the actions of PR at the systems network level.

In this study, pathway mapping result showed that the target genes of PR overlap more with Crohn's disease than with ulcerative colitis. Experimental study also suggested that the markers of both diseases are different from each other [59, 60]. The clinical symptoms of these diseases also differ; for instance, Crohn's disease affect any region of the entire gastrointestinal (GI) tract and all layers of the bowel wall, whereas ulcerative colitis affects only the mucosa and submucosa of colon [61]. Furthermore, ulcerative colitis can be cured by surgery, but Crohn's disease of any part of GI tract tends to relapse after surgery [62]. For this reason, long term management using herbal medicines might be highly recommendable treatment option for Crohn's disease, since herbal medicines have advantages for managing chronic diseases [63, 64].

In addition, IBD is usually referred to as an autoimmune disorder [65], whereas Crohn's disease does not meet the criteria of an autoimmune disorder; rather it is associated with immune deficiency or a secondary immune response to altered intestinal microbiota [65]. Furthermore, ulcerative colitis is a mucosal disease where autoimmune autoantibodies are commonly detected [66], whereas Crohn's disease is a transmural disease, in which pathological changes in gut wall are thought to result from submucosal inflammatory changes [67]. Accordingly, the areas targeted for treatment in these two diseases should be differentiated.

In the present study, we focused on the use of PR as a potential therapy for Crohn's disease. However, herb pairs and combinations are more commonly prescribed and are regarded to be more effective and safer [68]. In terms of the Gunsinjwasa theory of traditional medicine combinations, there are four different roles for each herb in the formula. First, the major component targets the main symptom. Second, the supportive component assists the effect of the major component or targets the secondary symptoms. Third, the neutralizing component allays the side effects or toxins of the major and the supportive component. Fourth, the deliver/retaining component guides the medicine to the targeting part of the body [69, 70]. This combination principle enables not only the enhancement of synergistic medicinal effects but also potentially reduces toxicities [69]. In order to induce better effects and reduce toxicities, an extended analysis of the* Pulsatillae Radix* (Baekduong, PR),* Phellodendri Cortex* (Hwangbaek),* Coptidis Rhizoma* (Hwangryeon), and* Citrus reticulata* (Jinpi) herb combination (a widely prescribed formula, known as Baekduong decoction) should be explored in the future.

Through GO analysis, we found out that targets of PR are associated with liver diseases such as hepatitis B, hepatitis C, and nonalcoholic fatty liver. In addition, the mRNA expression of 91 of 179 genes in liver was overexpressed according to the target organ location network result. Liver inflammation is as common extraintestinal symptom of Crohn's disease [71], and the number of liver abscesses in Crohn's disease patients is 15 to 20 times higher than that found in the general population [72]. Besides biochemical liver dysfunction [73] and hepatic fibrosis [74] are also frequently found in Crohn's disease, and a number of drugs used to treat IBD have been reported to be associated with liver injury [75]. Furthermore, in an experimental study using a mice model of Crohn's disease-like ileitis, it was found that TLR9 plays an important role in hepatic involvement in IBD [71]. More detailed pathways and relations between liver and Crohn's disease should be discussed in the future.

This study demonstrated that 73 and 67 of 158 targets of PR were highly expressed in small intestine and colon, respectively, results which were accordance with not only the organs commonly affected by Crohn's disease but also the properties of PR in terms of meridian tropism theory. However stronger evidence with another research design is required to support this result.

We confirmed a multicompound and multitarget interaction through the C-T network, which showed that 88% of the active compounds were connected with more than two targets and 47% of the targets interacted with more than one compound. Although it demonstrated the synergetic network of multitarget actions, we should explore more differentiated drug action based on degree centrality and how different the drug actions are when more compounds target one gene in simultaneous way compared to the case of one compound targeting one gene.

## 5. Conclusion

Previous studies indicate that most compounds in PR have anti-inflammatory, anticancer, and antioxidant effects. In the present study, we found that these compounds interact with multiple targets in a synergetic manner and that PR is highly connected to Crohn's disease related pathways, biological processes, and organs. C-T and T-P network results demonstrated complex multicompound and multitarget drug actions and the relations between targets and various pathways. Furthermore, target genes were found to be overexpressed in organs highly related to immunity. These findings suggest the anti-inflammatory effects of PR, and its enhancements of repair processes and immune system might be of therapeutic benefit in Crohn's disease.

## Supplementary Material

Supplementary Table S1 showed the ADME profiles of 57 compounds in PR, and supplementary Table S2 demonstrated the mRNA expression profiles of 179 of 182 genes.

## Figures and Tables

**Table 1:** 32 potential active compounds of *Pulsatillae Radix*.

ID	Active compounds	Structure	OB (%)	Caco-2	DL
C1	3-Methylcoumarin	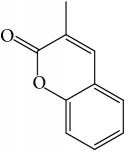	19.66	1.27	0.05

C2	5,6,7-Trimethoxycoumarin	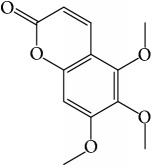	32.54	0.94	0.12

C3	AIDS045703	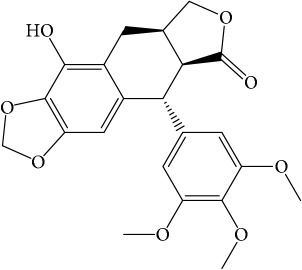	21.37	0.55	0.87

C4	Androstane-3,11,17-triol	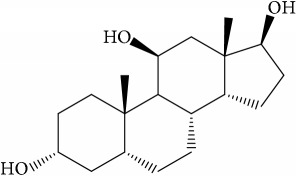	13.19	−0.04	0.38

C5	Anemosapogenin	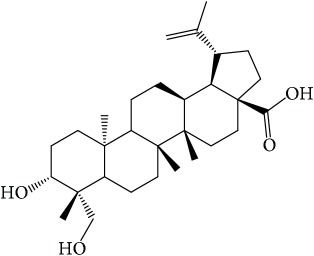	17.87	0.07	0.77

C6	Anemoside A3	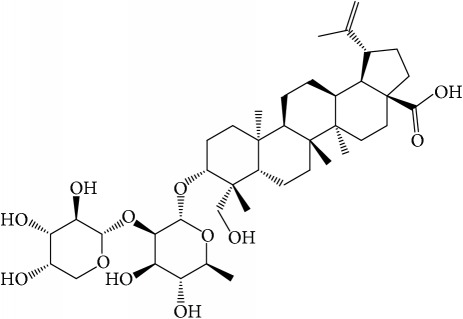	15.46	−1.6	0.15

C7	Aureusidin	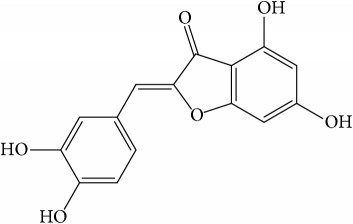	53.42	0.07	0.24

C8	Beta-sitosterol	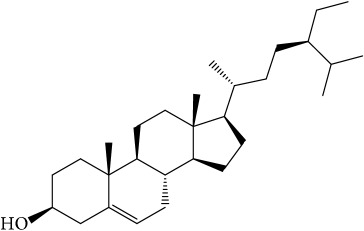	36.91	1.32	0.75

C9	Betulonic acid	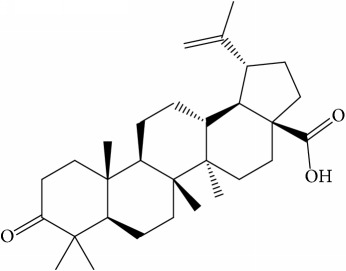	16.83	0.65	0.78

C10	Campesterol	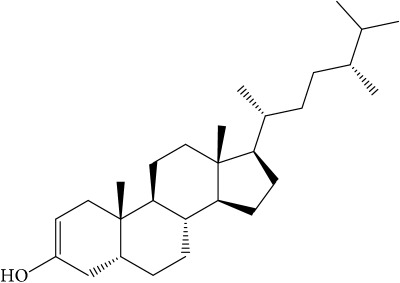	5.57	1.6	0.72

C11	Cauloside A	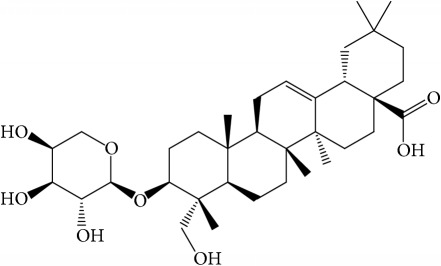	6.84	−0.82	0.4

C12	Cernuoside	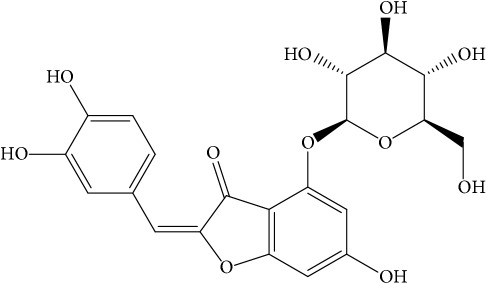	2.69	−1.51	−2.18

C13	Dauricine (8CI)	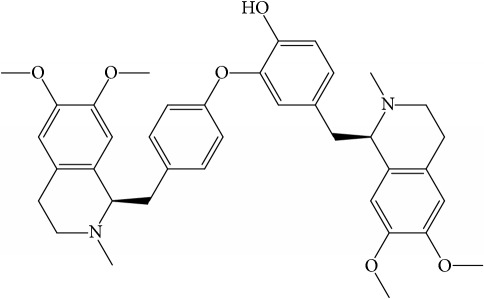	23.65	0.9	0.37

C14	Ergosterol	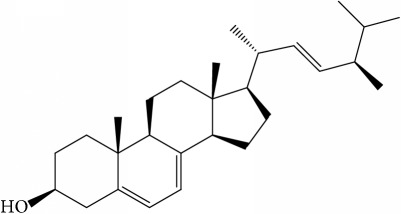	14.29	1.47	0.72

C15	Fraxinol	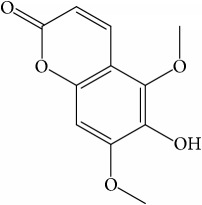	24.19	0.7	0.1

C16	Hederagenol	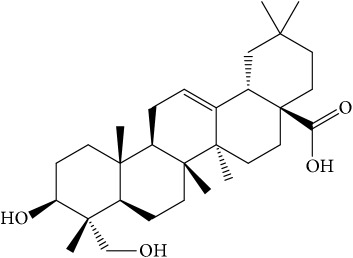	22.42	0.1	0.74

C17	Isorhamnetin	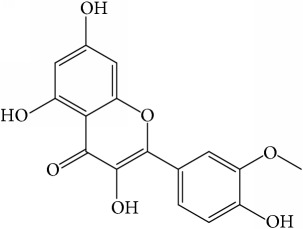	49.6	0.31	0.31

C18	LAN	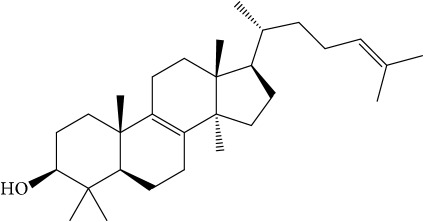	42.12	1.52	0.75

C19	Lignoceric acid	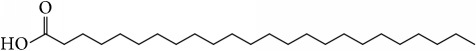	14.9	1.24	0.33

C20	Mairin	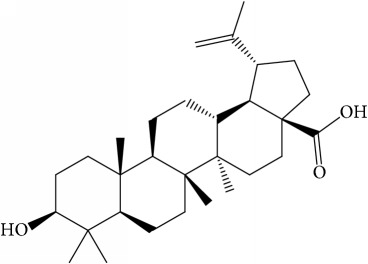	55.38	0.73	0.78

C21	Oleanolic acid	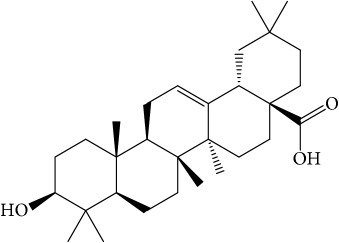	29.02	0.59	0.76

C22	Oleanolic acid deriv.	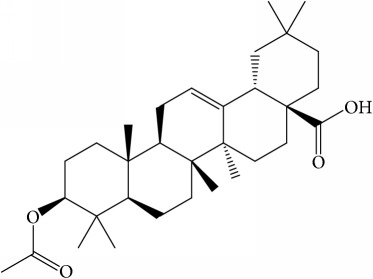	14.24	0.65	0.7

C23	Pinoresinol	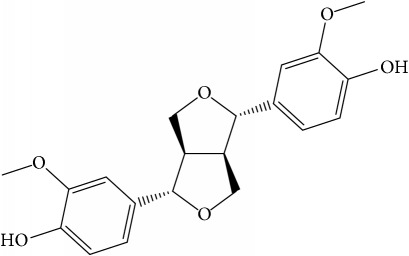	4.25	0.52	0.52

C24	Pulchinenoside A_qt	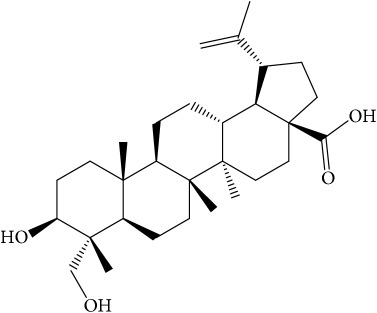	16.91	0.12	0.77

C25	Scoparone	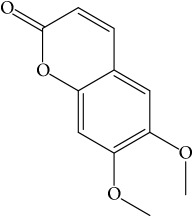	74.75	0.85	0.09

C26	Sitogluside	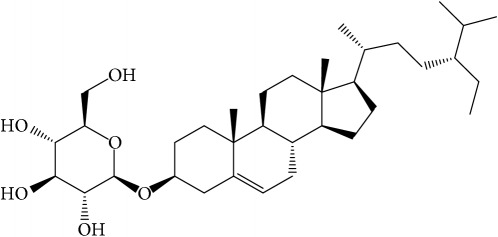	20.63	−0.14	0.62

C27	Sitosteryl acetate	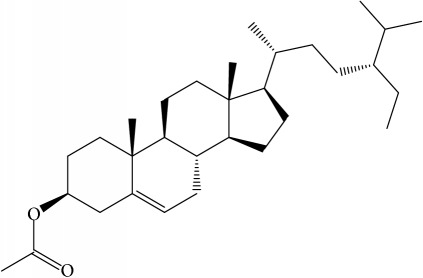	40.39	1.39	0.85

C28	Stigmasterol	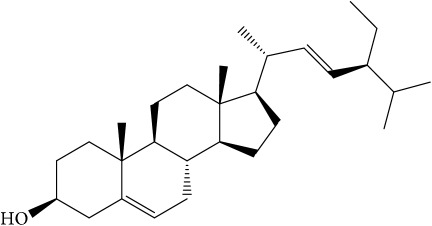	43.83	1.44	0.76

C29	Tricosanoic acid	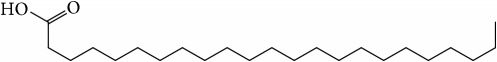	15.29	1.18	0.3

C30	Ursolic acid	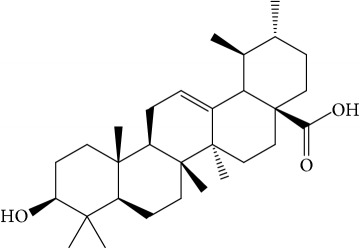	16.77	0.67	0.75

C31	ZINC01615307	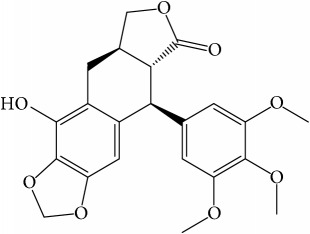	56.38	0.53	0.87

C32	*β*-Sitosterol	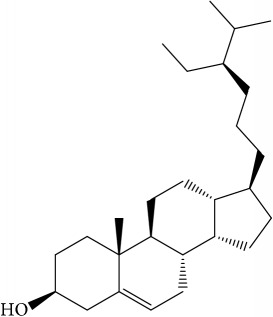	5.84	1.42	0.71

**Table 2:** Related targets of *Pulsatillae Radix*.

UniProt ID	Target	Gene name
Q92887	Canalicular multispecific organic anion transporter 1	ABCC2
P12821	Angiotensin-converting enzyme	ACE
P22303	Acetylcholinesterase	ACHE
P24666	Low molecular weight phosphotyrosine protein phosphatase	ACP1
P00326	Alcohol dehydrogenase 1C	ADH1C
P35348	Alpha-1A adrenergic receptor	ADRA1A
P35368	Alpha-1B adrenergic receptor	ADRA1B
P25100	Alpha-1D adrenergic receptor	ADRA1D
P08913	Alpha-2A adrenergic receptor	ADRA2A
P18825	Alpha-2C adrenergic receptor	ADRA2C
P08588	Beta-1 adrenergic receptor	ADRB1
P07550	Beta-2 adrenergic receptor	ADRB2
P15121	Aldose reductase	AKR1B1
O60218	Aldo-keto reductase family 1 member B10	AKR1B10
P09917	Arachidonate 5-lipoxygenase	ALOX5
P09923	Intestinal-type alkaline phosphatase	ALPI
P04746	Pancreatic alpha-amylase	AMY2A
P10275	Androgen receptor	AR
P15336	Cyclic AMP-dependent transcription factor ATF-2	ATF2
P05023	Sodium/potassium-transporting ATPase subunit alpha-1	ATP1A1
P15291	Beta-1,4-galactosyltransferase 1	B4GALT1
Q07812	Apoptosis regulator BAX	BAX
P10415	Apoptosis regulator Bcl-2	BCL2
Q07817	Bcl-2-like protein 1	BCL2L1
O15392	Baculoviral IAP repeat-containing protein 5	BIRC5
O43570	Carbonic anhydrase 12	CA12
Q8N1Q1	Carbonic anhydrase 13	CA13
P00918	Carbonic anhydrase II	CA2
P22748	Carbonic anhydrase IV	CA4
P35218	Carbonic anhydrase 5A, mitochondrial	CA5A
Q16790	Carbonic anhydrase VI	CA9
P62158	Calmodulin	CALM1
P29466	Caspase-1	CASP1
P42574	Caspase-3	CASP3
Q14790	Caspase-8	CASP8
P55211	Caspase-9	CASP9
P13500	C-C motif chemokine 2	CCL2
P20248	Cyclin-A2	CCNA2
P24385	G1/S-specific cyclin-D1	CCND1
P30279	G1/S-specific cyclin-D2	CCND2
P60033	CD81 antigen	CD81
P24941	Cell division protein kinase 2	CDK2
P11802	Cell division protein kinase 4	CDK4
Q00534	Cell division protein kinase 6	CDK6
P38936	Cyclin-dependent kinase inhibitor 1	CDKN1A
O14757	Serine/threonine-protein kinase Chk1	CHEK1
P11229	Muscarinic acetylcholine receptor M1	CHRM1
P08172	Muscarinic acetylcholine receptor M2	CHRM2
P20309	Muscarinic acetylcholine receptor M3	CHRM3
P08173	Muscarinic acetylcholine receptor M4	CHRM4
Q15822	Neuronal acetylcholine receptor subunit alpha-2	CHRNA2
P36544	Neuronal acetylcholine receptor protein, alpha-7 chain	CHRNA7
O15111	NF-kappa-B inhibitor alpha	CHUK
P16220	Cyclic AMP-responsive element-binding protein 1	CREB1
P15509	Granulocyte-macrophage colony-stimulating factor	CSF2RA
P17538	Chymotrypsinogen B	CTRB1
P07858	Cathepsin B	CTSB
P10145	Interleukin-8	CXCL8
Q16850	Lanosterol 14-alpha demethylase	CYP51A1
Q9UBM7	7-Dehydrocholesterol reductase	DHCR7
P27487	Dipeptidyl peptidase IV	DPP4
P21728	Dopamine D1 receptor	DRD1
Q9NRD8	Dual oxidase 2	DUOX2
Q6UWV6	Intestinal alkaline sphingomyelinase	ENPP7
Q99814	Endothelial PAS domain-containing protein 1	EPAS1
P03372	Estrogen receptor	ESR1
Q92731	Estrogen receptor beta	ESR2
P00742	Coagulation factor Xa	F10
P00734	Thrombin	F2
P08709	Coagulation factor VII	F7
P48023	Tumor necrosis factor ligand superfamily member 6	FASLG
P49327	Fatty acid synthase	FASN
P05230	Fibroblast growth factor 1	FGF1
P09038	Heparin-binding growth factor 2	FGF2
P01100	Proto-oncogene c-Fos	FOS
P14867	Gamma-aminobutyric acid receptor subunit alpha-1	GABRA1
P47869	Gamma-aminobutyric-acid receptor alpha-2 subunit	GABRA2
P34903	Gamma-aminobutyric-acid receptor alpha-3 subunit	GABRA3
P31644	Gamma-aminobutyric-acid receptor alpha-5 subunit	GABRA5
P17677	Neuromodulin	GAP43
Q8TDU6	G-protein coupled bile acid receptor 1	GPBAR1
P42262	Glutamate receptor 2	GRIA2
P49841	Glycogen synthase kinase-3 beta	GSK3B
Q9UII4	Probable E3 ubiquitin-protein ligase HERC5	HERC5
P09601	Heme oxygenase 1	HMOX1
P01112	GTPase HRas	HRAS
P28845	Corticosteroid 11-beta-dehydrogenase isozyme 1	HSD11B1
P80365	Corticosteroid 11-beta-dehydrogenase isozyme 2	HSD11B2
P08238	Heat shock protein HSP 90	HSP90AB1
P28223	5-Hydroxytryptamine 2A receptor	HTR2A
P46098	5-Hydroxytryptamine receptor 3A	HTR3A
P05362	Intercellular adhesion molecule 1	ICAM1
P01857	Ig gamma-1 chain C region	IGHG1
P01584	Interleukin-1 beta	IL1B
P60568	Interleukin-2	IL2
P05231	Interleukin-6	IL6
O15357	Phosphatidylinositol-3,4,5-trisphosphate 5-phosphatase 2	INPPL1
P05412	Transcription factor AP-1	JUN
Q12809	Potassium voltage-gated channel subfamily H member 2	KCNH2
Q12791	Calcium-activated potassium channel subunit alpha 1	KCNMA1
O75164	Lysine-specific demethylase 4A	KDM4A
P35968	Vascular endothelial growth factor receptor 2	KDR
Q99732	Lipopolysaccharide-induced tumor necrosis factor-alpha factor	LITAF
P09960	Leukotriene A-4 hydrolase	LTA4H
P21397	Amine oxidase [flavin-containing] A	MAOA
P27338	Amine oxidase [flavin-containing] B	MAOB
P11137	Microtubule-associated protein 2	MAP2
Q16539	Mitogen-activated protein kinase 14	MAPK14
P45983	Mitogen-activated protein kinase 8	MAPK8
Q13387	C-Jun-amino-terminal kinase-interacting protein 2	MAPK8IP2
Q07820	Induced myeloid leukemia cell differentiation protein Mcl-1	MCL1
P14174	L-Dopachrome tautomerase	MIF
P03956	Interstitial collagenase	MMP1
P09238	Stromelysin-2	MMP10
P08253	72 kDa type IV collagenase	MMP2
P08254	Stromelysin-1	MMP3
P14780	Matrix metalloproteinase-9	MMP9
P42345	Serine/threonine-protein kinase mTOR	MTOR
P14598	Neutrophil cytosol factor 1	NCF1
Q15788	Nuclear receptor coactivator 1	NCOA1
Q15596	Nuclear receptor coactivator 2	NCOA2
Q9GZQ4	Neuromedin-U receptor 2	NMUR2
P35228	Nitric oxide synthase, inducible	NOS2
P29474	Nitric oxide synthase, endothelial	NOS3
P15559	NAD(P)H dehydrogenase [quinone] 1	NQO1
P04150	Glucocorticoid receptor	NR3C1
P08235	Mineralocorticoid receptor	NR3C2
P01111	GTPase NRas	NRAS
P78380	Oxidized low-density lipoprotein receptor 1	OLR1
P35372	Mu-type opioid receptor	OPRM1
P07237	Protein disulfide-isomerase	P4HB
Q14432	CGMP-inhibited 3′,5′-cyclic phosphodiesterase A	PDE3A
P16284	Platelet endothelial cell adhesion molecule	PECAM1
P06401	Progesterone receptor	PGR
P42336	Phosphatidylinositol 4,5-bisphosphate 3-kinase catalytic subunit alpha isoform	PIK3CA
P48736	Phosphatidylinositol-4,5-bisphosphate 3-kinase catalytic subunit, gamma isoform	PIK3CG
P11309	Proto-oncogene serine/threonine-protein kinase Pim-1	PIM1
P61925	cAMP-dependent protein kinase inhibitor alpha	PKIA
P00749	Urokinase-type plasminogen activator	PLAU
P19174	1-Phosphatidylinositol 4,5-bisphosphate phosphodiesterase gamma-1	PLCG1
P09884	DNA polymerase alpha catalytic subunit	POLA1
P06746	DNA polymerase beta	POLB
P54098	DNA polymerase catalytic subunit	POLG
P27169	Serum paraoxonase/arylesterase 1	PON1
Q03181	Peroxisome proliferator activated receptor delta	PPARD
P37231	Peroxisome proliferator activated receptor gamma	PPARG
P17612	mRNA of PKA catalytic subunit C-alpha	PRKACA
P17252	Protein kinase C alpha type	PRKCA
P05129	Protein kinase C gamma type	PRKCG
P07477	Trypsin-1	PRSS1
P43115	Prostaglandin E2 receptor EP3 subtype	PTGER3
O14684	Prostaglandin E synthase	PTGES
P23219	Prostaglandin G/H synthase 1	PTGS1
P35354	Prostaglandin G/H synthase 2	PTGS2
P18031	Protein-tyrosine phosphatase 1B	PTPN1
P17706	T-cell protein-tyrosine phosphatase	PTPN2
P29350	Hematopoietic cell protein-tyrosine phosphatase	PTPN6
P11217	Glycogen phosphorylase, muscle form	PYGM
Q04206	Transcription factor p65	RELA
P19793	Retinoic acid receptor RXR-alpha	RXRA
Q14524	Sodium channel protein type 5 subunit alpha	SCN5A
P16581	E-selectin	SELE
Q9HAS3	Solute carrier family 28 member 3	SLC28A3
O43826	Glucose-6-phosphate translocase	SLC37A4
P13866	Sodium/glucose cotransporter 1	SLC5A1
P31639	Sodium/glucose cotransporter 2	SLC5A2
Q9NY91	Low affinity sodium-glucose cotransporter	SLC5A4
P23975	Sodium-dependent noradrenaline transporter	SLC6A2
Q01959	Sodium-dependent dopamine transporter	SLC6A3
P31645	Sodium-dependent serotonin transporter	SLC6A4
Q9Y6L6	Solute carrier organic anion transporter family member 1B1	SLCO1B1
P40763	Signal transducer and activator of transcription 3	STAT3
P59538	Taste receptor type 2 member 31	TAS2R31
P01137	Transforming growth factor beta-1	TGFB1
P01375	Tumor necrosis factor	TNF
P11388	DNA topoisomerase II alpha	TOP2A
P04637	Cellular tumor antigen p53	TP53
P17752	Tryptophan 5-hydroxylase 1	TPH1
P14679	Tyrosinase	TYR
P17643	5,6-dihydroxyindole-2-carboxylic acid oxidase	TYRP1
P15692	Vascular endothelial growth factor A	VEGFA
P47989	Xanthine dehydrogenase/oxidase	XDH

**Table 3:** Highly expressed targets in Crohn's disease.

UniProt ID	Target	Gene name
Q92887	Canalicular multispecific organic anion transporter 1	ABCC2
O60218	Aldo-keto reductase family 1 member B10	AKR1B10
P09917	Arachidonate 5-lipoxygenase	ALOX5
P62158	Calmodulin	CALM1
Q00534	Cell division protein kinase 6	CDK6
P10145	Interleukin-8	CXCL8
Q99814	Endothelial PAS domain-containing protein 1	EPAS1
P01100	Proto-oncogene c-Fos	FOS
P47869	Gamma-aminobutyric-acid receptor alpha-2 subunit	GABRA2
P49841	Glycogen synthase kinase-3 beta	GSK3B
P09601	Heme oxygenase 1	HMOX1
P05362	Intercellular adhesion molecule 1	ICAM1
P01584	Interleukin-1 beta	IL1B
P05231	Interleukin-6	IL6
P03956	Interstitial collagenase	MMP1
Q15788	Nuclear receptor coactivator 1	NCOA1
P35228	Nitric oxide synthase, inducible	NOS2
P01111	GTPase NRas	NRAS
P11309	Proto-oncogene serine/threonine-protein kinase Pim-1	PIM1
P61925	cAMP-dependent protein kinase inhibitor alpha	PKIA
P00749	Urokinase-type plasminogen activator	PLAU
P35354	Prostaglandin G/H synthase 2	PTGS2
P40763	Signal transducer and activator of transcription 3	STAT3
P17752	Tryptophan 5-hydroxylase 1	TPH1
